# Supplementary material for: Mesenchymal stem cells offer a drug-tolerant and immune-privileged niche to Mycobacterium tuberculosis
Source: Nat Commun. 2020 Jun 16;11:3062. doi: 10.1038/s41467-020-16877-3 (PMC7297998; doi:10.1038/s41467-020-16877-3)

Supplementary text

Mesenchymal stem cells offer a drug-tolerant and immune-privileged niche to *Mycobacterium tuberculosis*

Jain et al.

Corespondance: **dhiraj@icgeb.res.in**

Contains legends for:

Supplementary figures: 1-7

Supplementary Data: 1

Supplemental Figure 1: Characterization of ADSC and its differentiation potential

(A) Line histogram of ADSCs stained with CD73, CD44, CD105, CD90 and CD271 (blue) and corresponding isotype controls (Red). **(B)** Dot plot of ADSCs costained with CD73-FITC and CD271-Alexa fluor 647, only Alexa fluor 647 - secondary control and CD73-FITC with CD11b-PE. **(C)** Uninfected ADSCs differentiated into osteocytes, using specific differentiation media according to the manufacturer's protocol and stained with safranin O. Cells were visualized under light microscope at 10X. These differentiated cells were analyzed for osteocyte lineage specific genes using real time PCR. Similarly, 6th day GFP- H37Rv infected ADSCs were stained with safranin O and visualized under fluorescence and light microscope (Scale bar: 100µm). Parallely, they were processed for specific genes for RT-PCR. Mean ± SD, n= 3 **(D)** Uninfected ADSCs differentiated into chondrocytes, using specific differentiation media according to the manufacturer's protocol and stained with Alizarin Red S. Cells were visualized under light microscope at 10X (Scale bar: 100µm). These differentiated cells were analyzed for chondrocyte lineage specific genes using real time PCR. Similarly staining and RT-PCR was carried out in 6th day *GFP- H37Rv* infected ADSCs. Mean ± SD, n= 3 **(E)** Uninfected ADSCs were differentiated into Adipocytes, using specific differentiation media according to the manufacturer's protocol and stained with Oil Red O. Cells were visualized under light microscope at 40X (Scale bar: 20µm). Additionally, undifferentiated (Blue histogram) and differentiated (Orange histogram) cells were also analyzed for lipid accumulation using LipidTox staining by flow cytometry. Oil Red O staining and LipidTox staining were also carried out in 6th day *GFP- H37Rv* infected ADSCs. Blue histogram represent uninfected ADSC while orange histogram represent6th day infected ADSCs. Source data is included in Source data File.

Supplementary figure 1

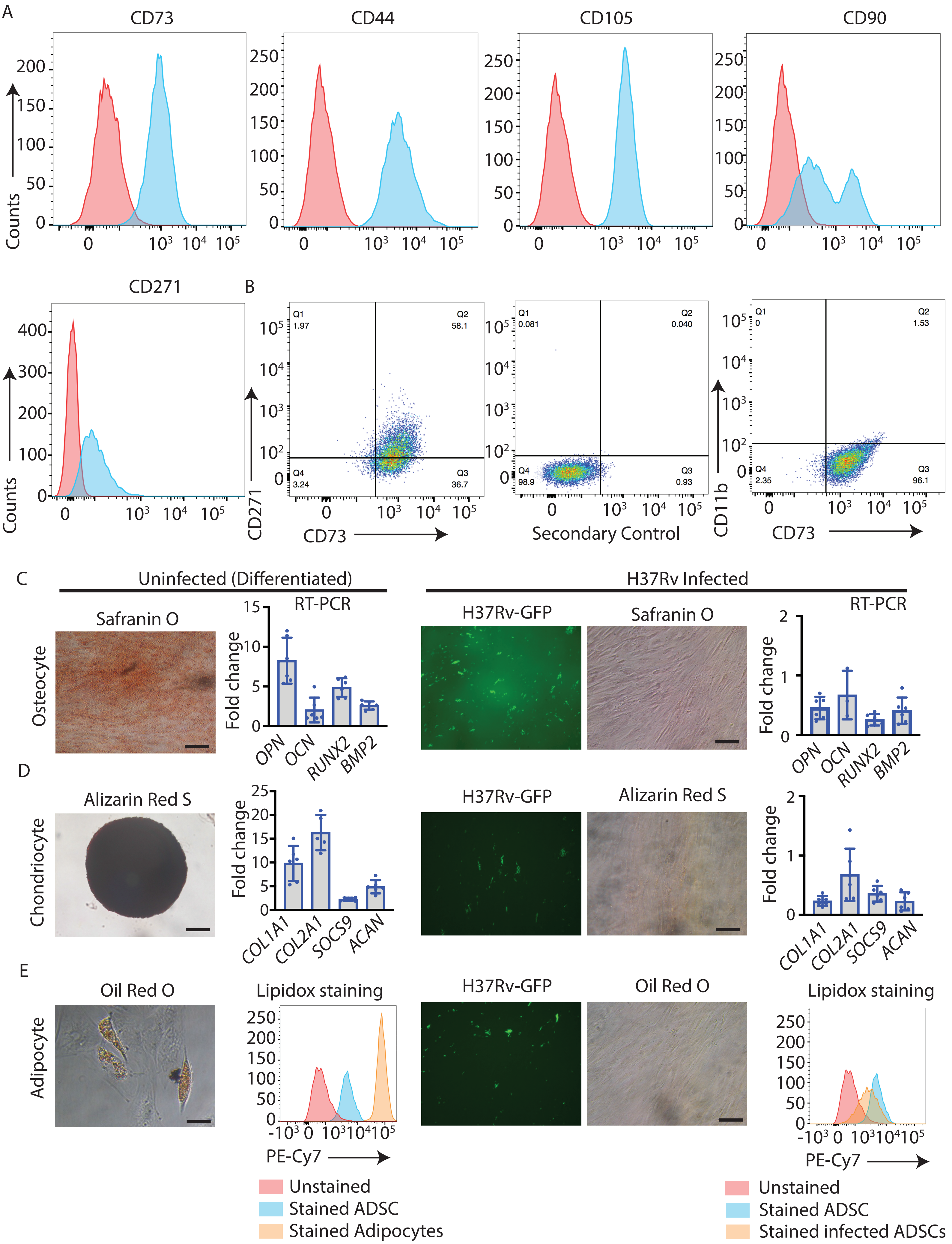

Supplemental Figure 2: Infection of ADSCs with H37Rv

(A) Volcano plot depicting differentially regulated genes (Upregulated in red, downregulated in green) in ADSCs upon *Mtb* infection with respect to uninfected ADSCs with p-value at y-axis and log_2 fold change on x-axis. In circular visualization, each pie represents a significantly enriched biological process. Outer circle represents scatter plot of the genes differentially regulated in each biological process. Red dots represents up-regulated genes while green dots represent down-regulated genes. The size of the inner circle represents adjusted p-value. The color of the inner circle represents z-score which indicates if a biological process is overall increased or decreased. Description of each process is detailed in the table. **(B)** Percentage drug tolerant population to 3 different doses of INH (0.5, 1 and 5 μg/ml) and RIF (0.1, 0.5 and 1 μg/ml) across 4 different time points (3rd , 6th , 9th and 12th day) in *H37Rv* infected ADSCs. Data is represented as mean ± SD, n = 3 donars. Source data is included in Source data File.

Supplementary figure 2

A

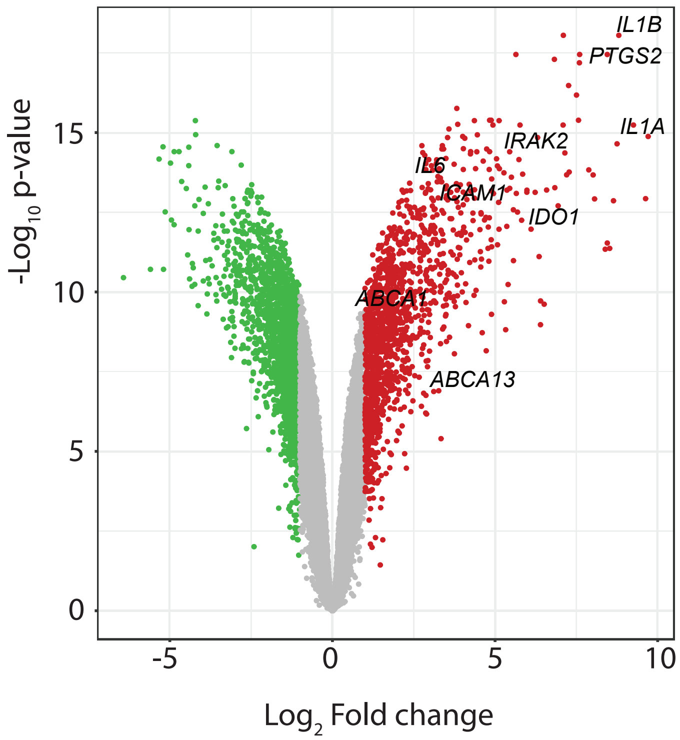

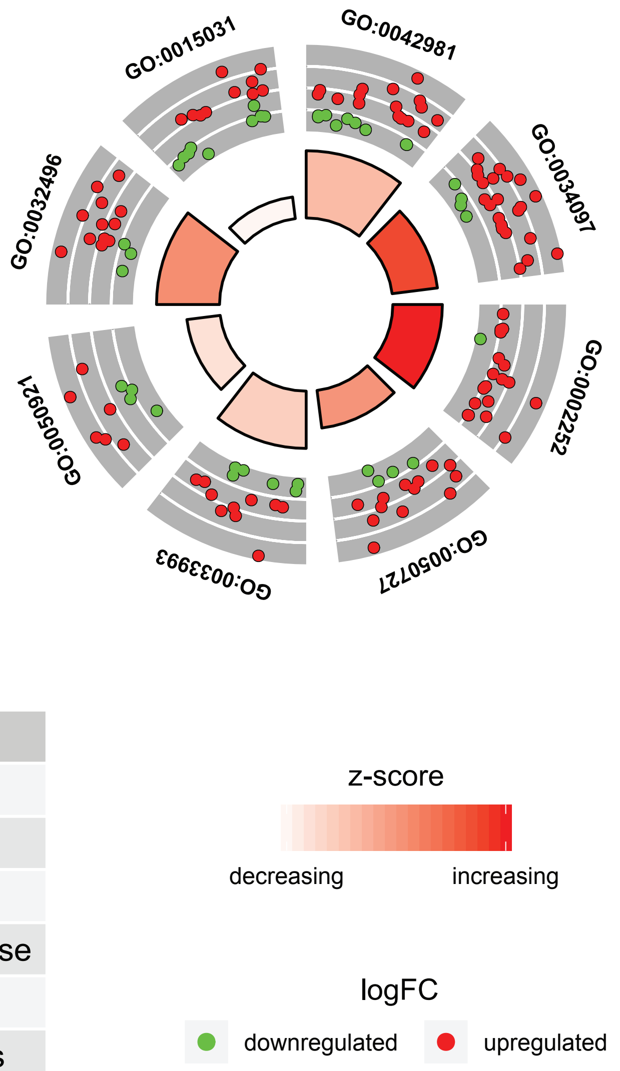

ID	Description
GO:0042981	Regulation of apoptotic process
GO:0034097	Response to cytokine
GO:0002252	Immune effector process
GO:0050727	Regulation of inflammatory response
GO:0033993	Response to lipid
GO:0050921	Positive regulation of chemotaxis
GO:0032496	Response to lipopolysaccharide
GO:0015031	Protein transport

B

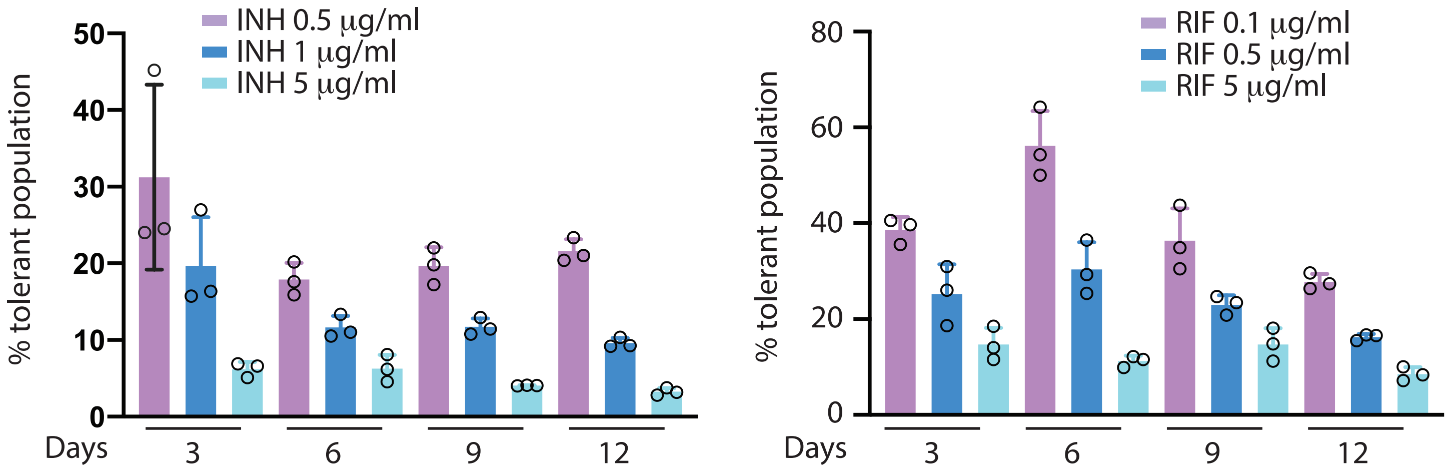

Supplemental Figure 3: Role of efflux pumps ABCC1 and ABCG2 in determining bacterial phenotypes within ADSCs

(A) Fold change in mRNA expression of *ABCC1* and *ABCG2* in ADSCs infected with different MOI of bacteria i.e.1, 5, 10, 25 and 50 on 6th day post-infection as determined by qPCR. *β-tubulin* was used as a constitutive housekeeping control and data was normalized to uninfected ADSCs. Mean ± SD, n = 3 **(B-C)** Percent INH tolerant *H37Rv* population in ADSCs after addition of novobiocin (25 µg/ml) (n = 2, with 5 independent observations each) **B**) or doses of MK571 , 10, 25, 50 µM (n = 4, **C**) for 24 hours before CFU plating on 6th day post-infection. **(D-E)** *In vitro* growth of *H37Rv* broth culture in the presence of novobiocin (10, 25, 50, 75, 100 µg/ml) (D) or MK571 (10,25,50,75,100 µM) (E) along with vehicle control (DMSO) after 24 hours of incubation and measured spectrophotometrically (O.D. 600 nm). Mean ± SD, n = 3 **(F)** Line histogram of intracellular (I.C.) and surface expression of ABCC1/MRP-1 and ABCG2/BCRP in uninfected and 6th day *H37Rv* infected ADSCs with and without respective siRNA knockdowns. Numbers represent MFI of individual histogram. **(G-H)** CFU assay of *Mtb* burden on 6th day in *H37Rv* infected ADSCs after 24 hours prior treatment with different doses of MK571 (G, Mean ± SD, n = 3-5) and 25 µg/ml of novobiocin (H, Mean ± SD, n = 2, 5 independent observations each), Data were analyzed using unpaired two tailed Student's t-test (A, B, D, E, G, H) and one-way ANOVA (C). ****P < 0.0001, NS 'not significant' Source data are included in the Source data File.

Supplementary figure 3

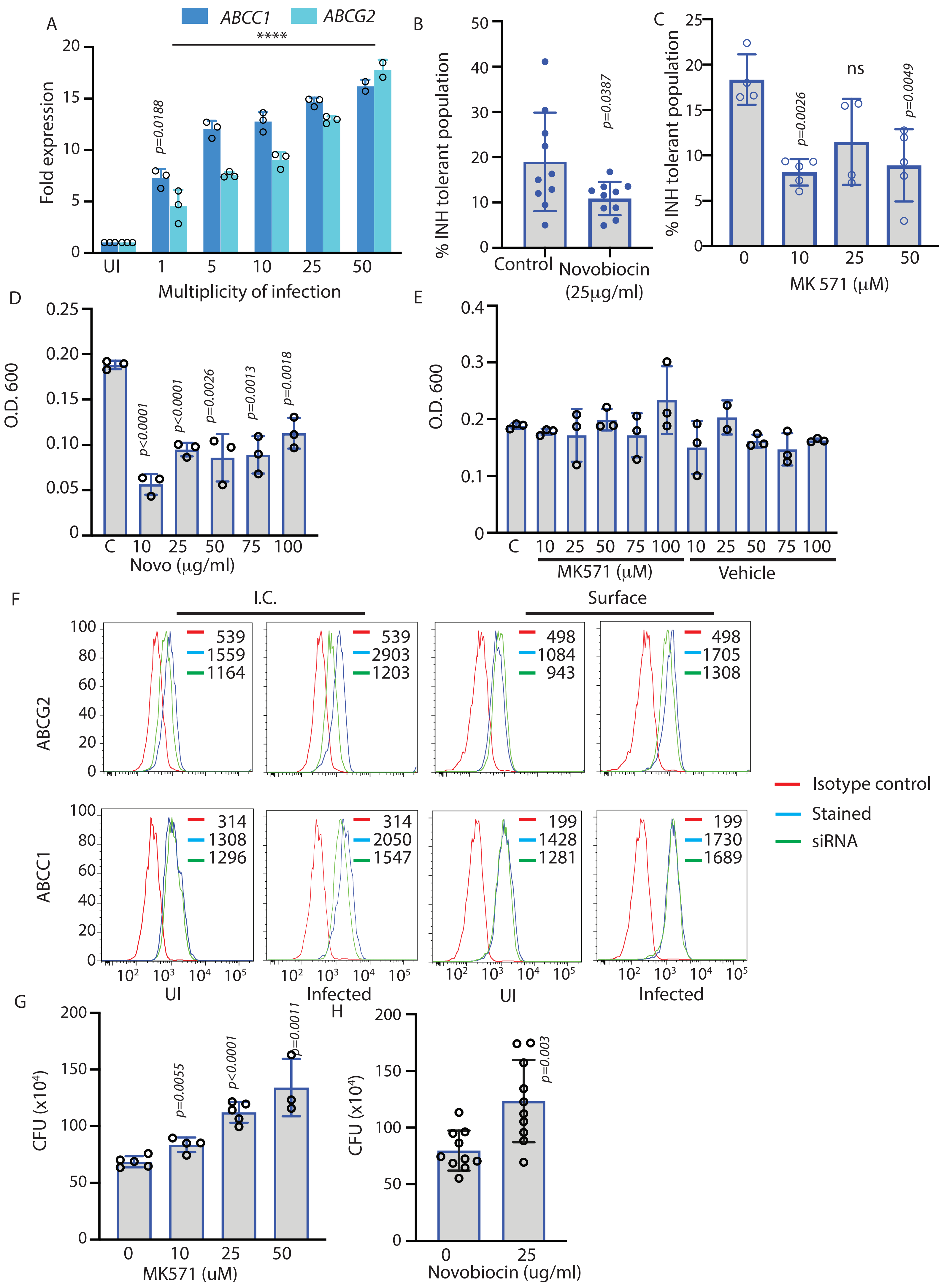

Supplemental Figure 4: Intracellular niches of H37Rv within ADSCs

(A-B) Representative confocal images of PKH67 labeled *H37Rv* (green) infected ADSCs stained with Rab5 (red) along with DAPI (A) and Rab7 (red) with DAPI (B) 3rd day post-infection. Bar graphs at the right shows percent localization of *Mtb* with Rab5 and Rab7 compartments respectively. Mean ± SEM, n = 5-6 independent experiments **(C)** Percent localization of PKH67 labeled *H37Rv* to Rab5 and Rab7 compartments in THP1 macrophages at 48 hours post-infection. Bar graph represent mean ± SEM, n = 3-4 independent experiments **(D)** Confocal images of PKH67 labelled *H37Rv* infected ADSCs (green) stained for lysosomal markers i.e. LAMP-1 (blue), Cathepsin D (orange) and LysoTracker Red after 24 hour incubation with IFNγ (5 ng/ml), TNFα (20 ng/ml) or MK571 (50 µM) or left untreated prior to fixation on 3rd day post-infection. **(E)** PKH67 labeled *H37Rv* infected ADSCs stained with ABCC1 or ABCG2 (orange) along with LysoTracker red. Scale bars, 10 µm. For D, E images are representative of 3 to 5 independent experiments. Source data are included in the Source data File.

Supplementary figure 4

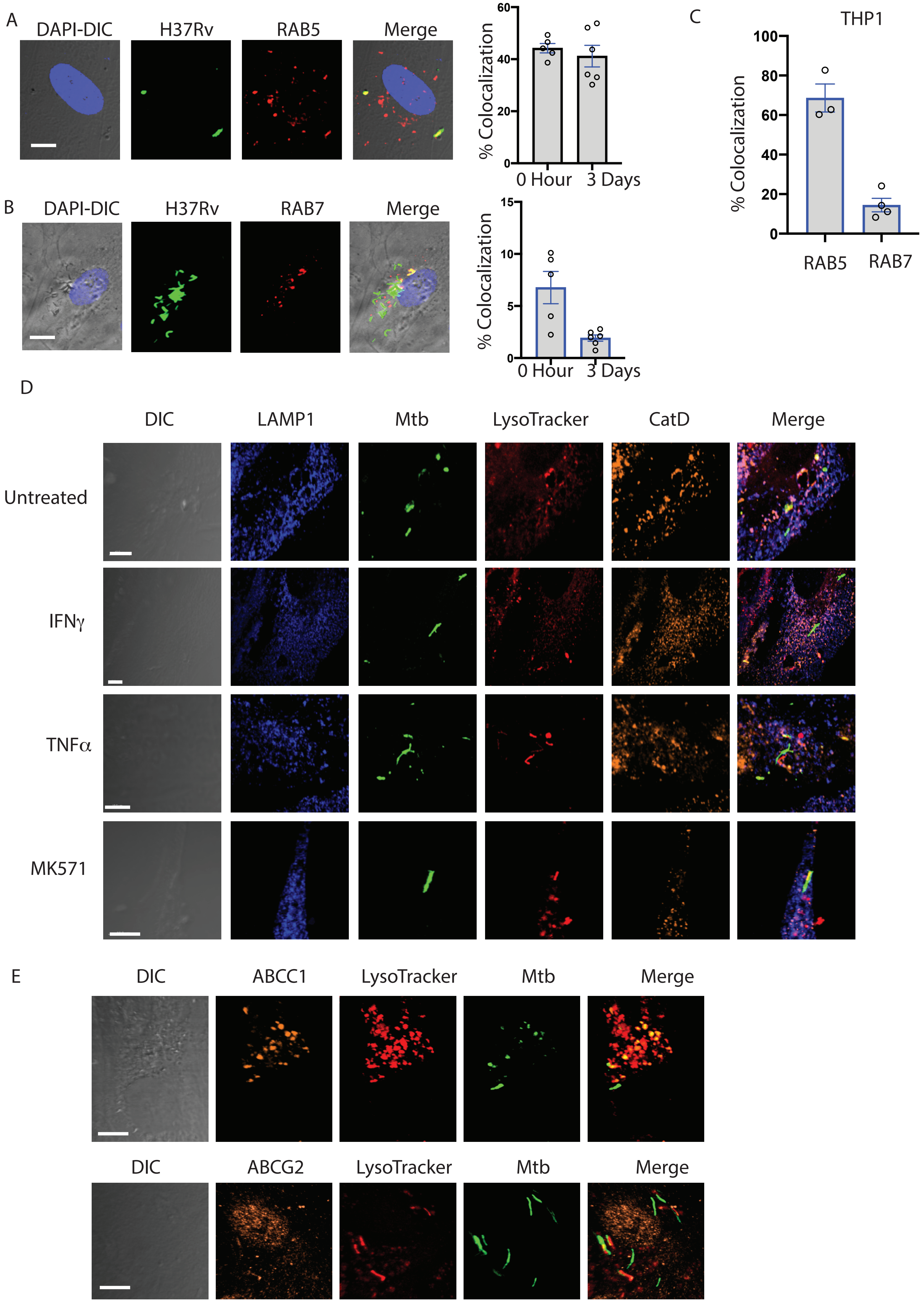

Supplemental Figure 5: Autophagy regulation in ADSCs upon *Mtb* infection and immune activation

(A) Representative confocal images of *GFP-H37Rv* infected ADSCs stained with LC3 (red) and DAPI (blue) without or with the addition of BafA1 (100 nM) for 3 hours, 3rd day post-infection. Bar graph at the right represents percent localization of *Mtb* with LC3 compartment on Day 0 and Day 3 (top) and on addition of BafA1 (bottom). Bar graph represent mean ± S.E.M., n = 4-7 independent experiments **(B)** Immunoblot of uninfected ADSCs probed for LC3 protein with GAPDH as loading control after addition of rapamycin (100 nM, 6 hours) and starvation/HBSS (6 hours) in the absence or presence of BafA1 (100 nM, 3 hours). Immunoblot for LC3 was also performed for uninfected and 6th day *H37Rv* infected ADSCs with and without BafA1 (100 nM, 3 hours) with GAPDH as loading control. Image is representative of 4 independent experiments **(C)** Immunoblot of uninfected and 6th day *H37Rv* infected ADSCs probed for LC3 protein and GAPDH upon 24 hours treatment with 5 ng/ml IFNγ and 20 ng/ml TNF with and without the addition of BafA1 (100 nM, 3 hours). Image is representative of 2 independent experiments **(D)** Line histogram of cellular ROS measured using DCFDA dye (5 μM, 30 minutes) within uninfected and *H37Rv* infected ADSCs left untreated or treated with increasing doses of IFNγ (5, 12.5 and 25 ng/ml). Immunoblotting and FACS experiments were repeated thrice independently. Scale bar, 10 μm. Source data is included in Source data File.

Supplementary figure 5

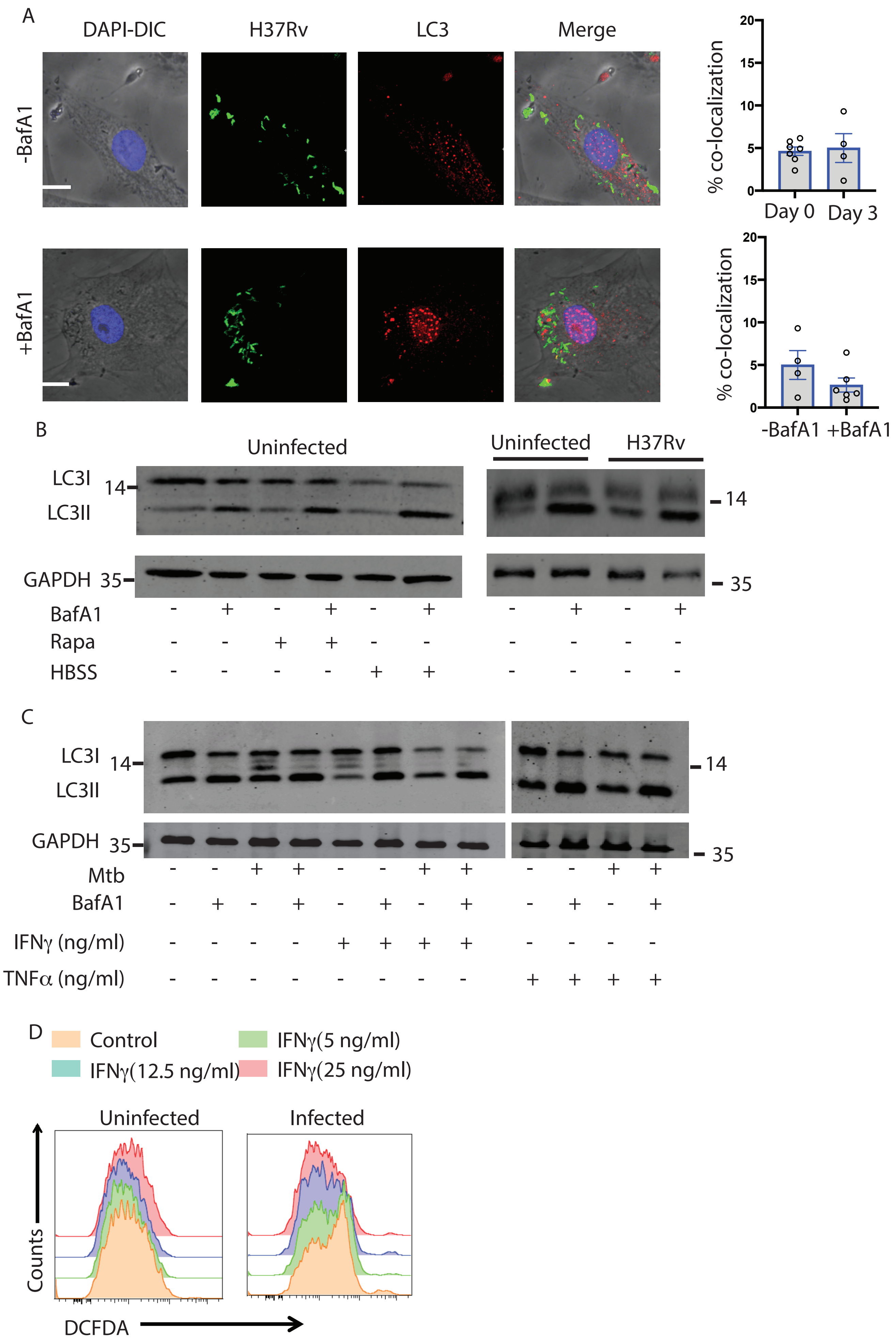

Supplemental Figure 6: PGE2 plays key role in providing the protective niche to *Mtb* within ADSCs

(A) Bar graph depicts the log_2 fold change in the expression of genes known for MSC-mediated immunomodulation, in ADSCs on 6th day post-infection from the microarray analysis (Supplementary Data 1). **(B)** CFU burden of 6th day infected ADSC when left untreated or treated with IFNγ, TNFα and MK571 along with increasing doses of PF04418948 (50, 250 and 500 nM). Data shown is mean ± SD, n = 5 **(C)** CFU assay in infected THP-1 macrophages after addition of increasing dose of celecoxib (50, 150, 250 μM) for 24 hours prior to 3rd day plating. Mean ± SD, n = 5 **(D)** PGE2 ELISA of supernatants from uninfected and *H37Rv* infected THP-1 macrophages collected on 3rd day post-infection, which were also untreated or treated with IFNγ (5 ng/ml), TNFα (20 ng/ml), and celecoxib (250 μM) for 24 hours, performed according to the manufacturer's protocol. (n = 2 independent experiments, one observation each) **(E)** Immunoblot showing knockdown of Cox-2 protein 48 hours after transfecting 100 nM and 200 nM of cox-2 siRNA or scrambled control. GAPDH is used as a loading control. **(F)** Representative confocal images of PKH67 labelled *H37Rv* infected ADSCs dual stained with LAMP-1 and CatD along with LysoTracker red after treatment with celecoxib (250 μM, 24 hours) or PF (500 nM, 24 hours) or cox-2 siRNA (100 nM , 48 hours) 3rd day post-infection. Scale bars, 10 μm. Data is analyzed by unpaired two tailed t-test (A, C) and one-way ANOVA (B) Source data is included in Source data File.

Supplementary figure 6

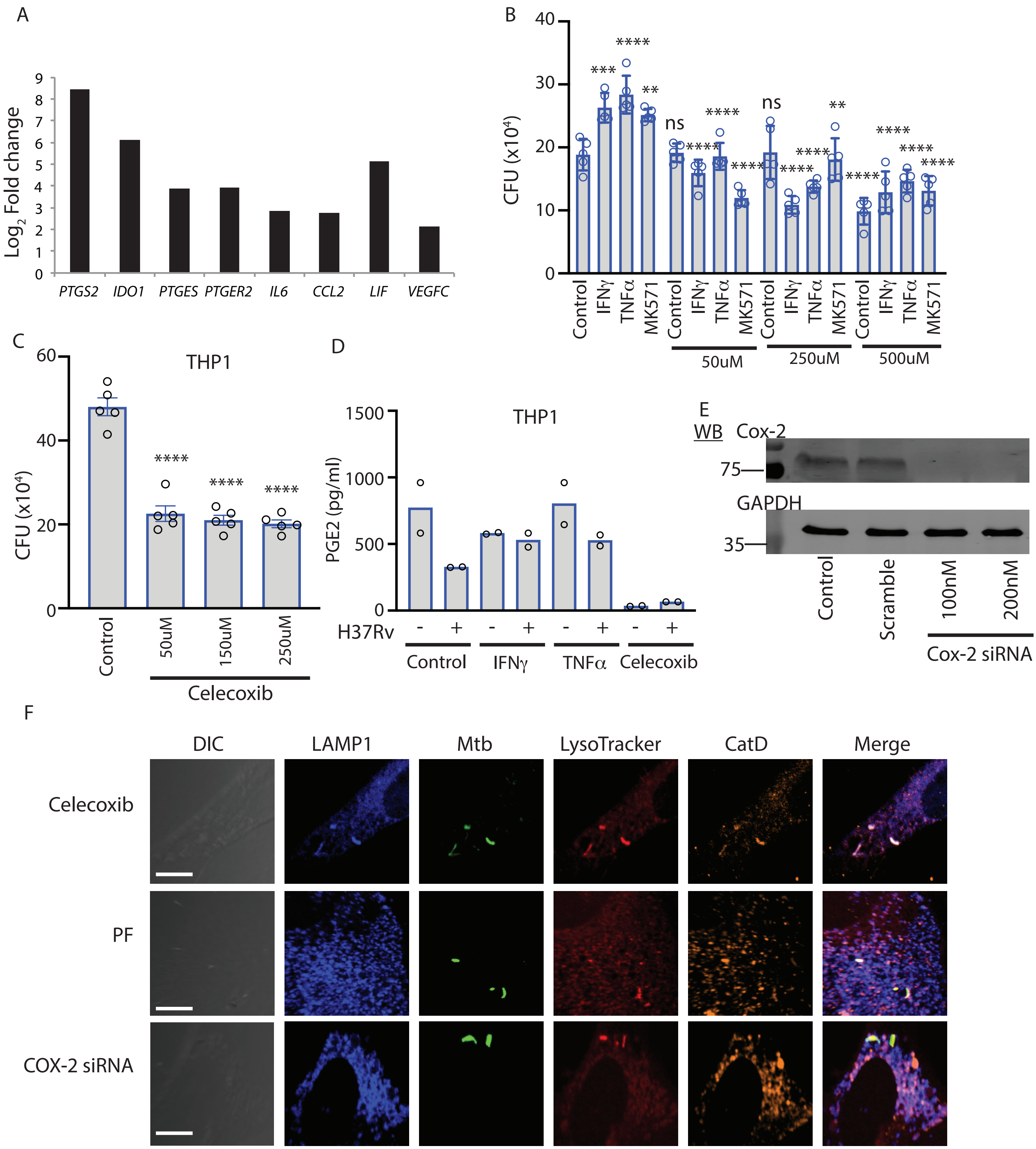

Supplemental Figure 7: *Mtb*-MSC colocalization *in vivo*.

(A) Percentage purity of sorted MSC and macrophage population from the lungs of *H37Rv* infected mice. (B) Plot showing percent macrophage and MSC population in the lungs of uninfected animals (green) with respect to those in the *Mtb* infected ones (purple). The data from the infected animals are same as shown in Fig. 4 (n=10). For uninfected animals, n=3 for data at week 4 and n = 2 for data at week 12. (C) For antibody controls, skin biopsy from a patient with known *Lupus vulgaris* infection was included. The left panel show isotype controls with no brown Ag85B and blue CD73 positivity. Scale bar, 100 µm. Fast red stain was used to highlight the background stromal cells. The right panel shows Ag85B positive organism studded histiocytes (brown positivity) and surrounding CD73 positive mesenchymal cells (blue color) (x 100, scale bar = 20 µm). Panel D from left to right shows isotype control used, positive control used for two colored immunofluorescence staining using Ag85B (green) with CD73 stains (red), and Ag85B (green) with CD105 (red) stains (x 100). Scale bar, 100 µm.

Supplementary figure 7

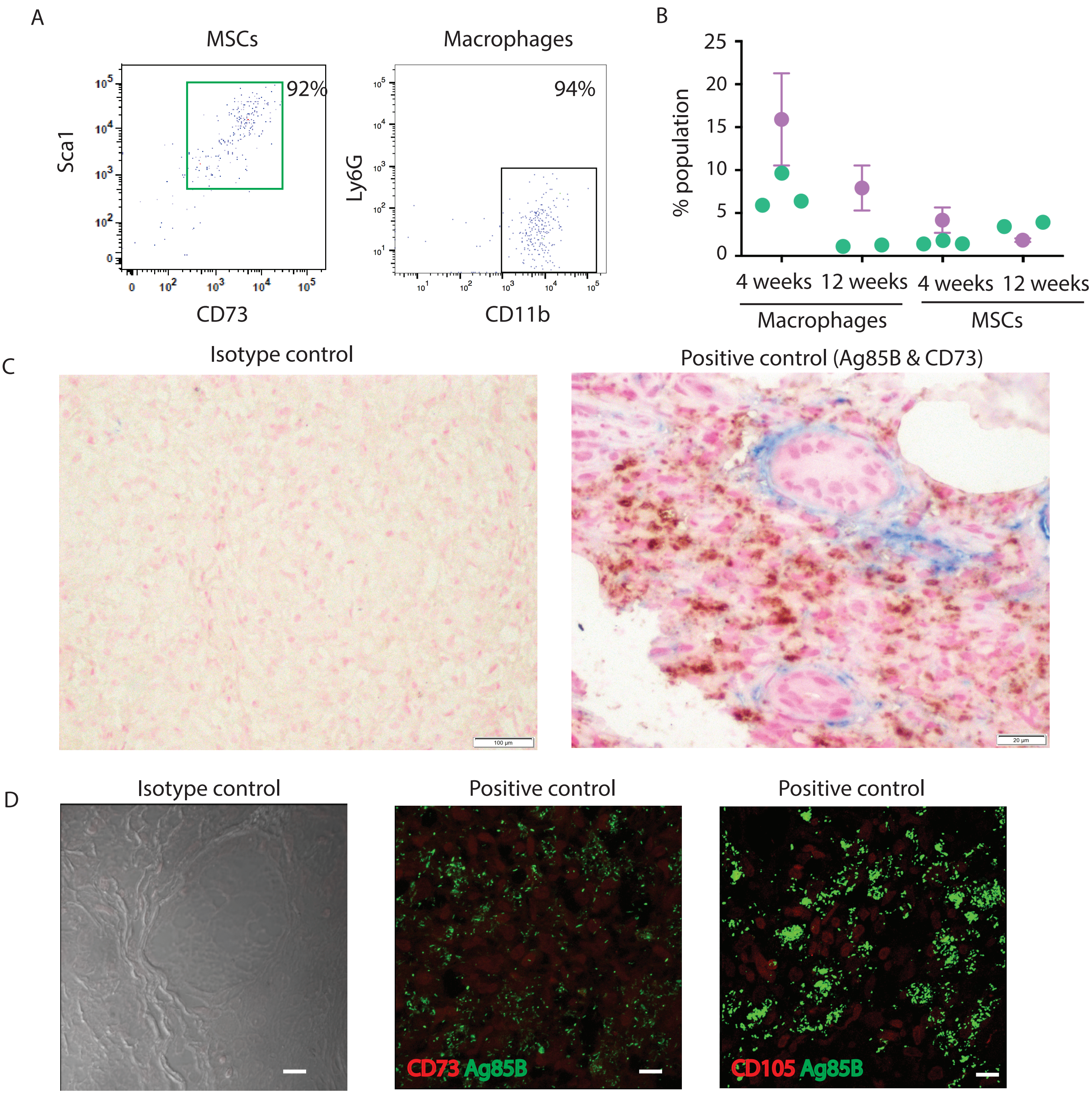

Supplement: Supplementary file 1 — Supplementary Information [file 41467_2020_16877_MOESM1_ESM.pdf]
