# Supplementary material for: Mesenchymal stem cells offer a drug-tolerant and immune-privileged niche to Mycobacterium tuberculosis
Source: Nat Commun. 2020 Jun 16;11:3062. doi: 10.1038/s41467-020-16877-3 (PMC7297998; doi:10.1038/s41467-020-16877-3)
Supplement: Supplementary file 3 — Reporting Summary [file 41467_2020_16877_MOESM3_ESM.pdf]

nature research

Corresponding author(s): Dhiraj Kumar

Last updated by author(s): 5/25/2020

# Reporting Summary

Nature Research wishes to improve the reproducibility of the work that we publish. This form provides structure for consistency and transparency in reporting. For further information on Nature Research policies, see Authors & Referees and the Editorial Policy Checklist .

## Statistics

For all statistical analyses, confirm that the following items are present in the figure legend, table legend, main text, or Methods section.

| n/a | Confirmed | |
|---|---|---|
| ☐ | ☒ | The exact sample size (*n*) for each experimental group/condition, given as a discrete number and unit of measurement |
| ☐ | ☒ | A statement on whether measurements were taken from distinct samples or whether the same sample was measured repeatedly |
| ☐ | ☒ | The statistical test(s) used AND whether they are one- or two-sided<br>*Only common tests should be described solely by name; describe more complex techniques in the Methods section.* |
| ☒ | ☐ | A description of all covariates tested |
| ☒ | ☐ | A description of any assumptions or corrections, such as tests of normality and adjustment for multiple comparisons |
| ☐ | ☒ | A full description of the statistical parameters including central tendency (e.g. means) or other basic estimates (e.g. regression coefficient) AND variation (e.g. standard deviation) or associated estimates of uncertainty (e.g. confidence intervals) |
| ☐ | ☒ | For null hypothesis testing, the test statistic (e.g. *F*, *t*, *r*) with confidence intervals, effect sizes, degrees of freedom and *P* value noted<br>*Give P values as exact values whenever suitable.* |
| ☒ | ☐ | For Bayesian analysis, information on the choice of priors and Markov chain Monte Carlo settings |
| ☒ | ☐ | For hierarchical and complex designs, identification of the appropriate level for tests and full reporting of outcomes |
| ☒ | ☐ | Estimates of effect sizes (e.g. Cohen's *d*, Pearson's *r*), indicating how they were calculated |

*Our web collection on statistics for biologists contains articles on many of the points above.*

## Software and code

Policy information about availability of computer code

| | |
|---|---|
| Data collection | FACSDIVA (BD), NIS Elements (Nikon) |
| Data analysis | Imaris X 64 7.6.4 (Bitplane), Image-J 1.48, NIS Elements AR 3.2 (Nikon), FlowJo 10.5.3 (BD), GraphPad Prism Version 8.0, Miscrosoft Excel 2011, R packages (GenomeStudio, beadarray, genefilter, limma) |

For manuscripts utilizing custom algorithms or software that are central to the research but not yet described in published literature, software must be made available to editors/reviewers. We strongly encourage code deposition in a community repository (e.g. GitHub). See the Nature Research guidelines for submitting code & software for further information.

## Data

Policy information about availability of data

All manuscripts must include a data availability statement. This statement should provide the following information, where applicable:

- Accession codes, unique identifiers, or web links for publicly available datasets
- A list of figures that have associated raw data
- A description of any restrictions on data availability

The raw files for the microarray experiment are available at GEO database “GSE133803[https://www.ncbi.nlm.nih.gov/geo/query/acc.cgi?acc=GSE133803]”. For all the plots in figures 1, 2, 3, 4 and supplementary figures 1,2,3,4,5,6, 7B, corresponding raw data are provided in the Source Data File. Any additional relevant data is available on request.

# Field-specific reporting

Please select the one below that is the best fit for your research. If you are not sure, read the appropriate sections before making your selection.

# Life sciences study design

All studies must disclose on these points even when the disclosure is negative.

| | |
|---|---|
| Sample size | It is accepted norm in the filed to perform all experiments in at least three independent biological replicates. Through out the manuscript, all the experiments were performed with n=3 or n>3 (explicitly mentioned for each dataset). For experiments, like PGE2 measurement in ADSCs and effect of COX2 inhibition on bacteriual CFU, ADSCs from three independent donors were used. For each donor, experiments were repeated at least for three independent experiments. Microarray experiments were peformed on three replicates, which is again an accepted norm for medium throughput experiments. Experiments on mice were repeated three times with total n=10. |
| Data exclusions | No data were excluded from the analyses. |
| Replication | All experiments were done thrice independently. In each experiment n =3 samples were carried on unless otherwise mentioned. Data from all completed replicates are included in the analysis. |
| Randomization | All samples or mice in experiments were randomized irrespective of the treatment. |
| Blinding | While there was no pre-defined blinding, most experiments were performed by multiple people at different stages (sample preparation, data acqusition and analysis), thereby nullyfying the chances of bias. For animal experiments, treatment groups were not revealed to the person responsible for giving the treatments. |

# Reporting for specific materials, systems and methods

We require information from authors about some types of materials, experimental systems and methods used in many studies. Here, indicate whether each material, system or method listed is relevant to your study. If you are not sure if a list item applies to your research, read the appropriate section before selecting a response.

## Materials & experimental systems

| n/a | Involved in the study | |
|---|---|---|
| ☐ | ☒ | Antibodies |
| ☐ | ☒ | Eukaryotic cell lines |
| ☒ | ☐ | Palaeontology |
| ☐ | ☒ | Animals and other organisms |
| ☐ | ☒ | Human research participants |
| ☒ | ☐ | Clinical data |

## Methods

| n/a | Involved in the study | |
|---|---|---|
| ☒ | ☐ | ChIP-seq |
| ☐ | ☒ | Flow cytometry |
| ☒ | ☐ | MRI-based neuroimaging |

## Antibodies

| | |
|---|---|
| Antibodies used | MAP1LC3B, (Novus, NB100-2220), GAPDH (Santa Cruz Biotechnology, sc-48167), Cox-2 (Abcam, ab62331), Rab5 (Santa Cruz Biotechnology, sc-309), ABCC1 (Santa Cruz Biotechnology, sc-18835), Rab7 (Santa Cruz Biotechnology, sc-6563), LAMP-1 (Santa Cruz Biotechnology, sc-20011), ABCG2 (Santa Cruz Biotechnology, sc-58222), CathepsinD (Abcam, ab19555), CD73-FITC (clone AD2, BD 561254), CD11b-PE(BD 557321), CD271 (Abcam,ab8874), CD44 (Thermoscientific, MS668P), CD90 (Abcam, ab181469) and CD105 (Abcam, ab114052). CD45-APC (clone 30-F11; 559864, BD Bioscience), CD73-BV450 (clone TY/23; 561544, BD Bioscience), CD11b-APC-Cy7 (clone M1/70; 557657, BD Bioscience), Ly6G-PE (clone 1A8; 551461, BD Bioscience), Sca-1-PE-CF594 (clone D7; 562730, BD Bioscience), CD90-BB515 (clone OX7, 564607, BD Bioscience), CD44-BV605 (Clone IM7, 563058, BD Bioscience), CD105-PE (Clone MJ7/18, 562759, BD Bioscience), Ly6c-BV605 (Clone AL-21, 563011, BD Bioscience), I-A/I-E-BB700 (clone 2G9, 746086,BD Bioscience), CD11c-PE-Cy7 (cloneHL3, 558079, BD Bioscience), Rabbit anti-Ag85B (ab43019, abcam), Mouse anti-CD73 (1D7, ab91086, abcam), Mouse anti-105 (SN6, ab11414, abcam), Rabbit IgG1 polyclonal Isotype control (ab37415, abcam), Mouse IgG1 kappa monoclonal Isotype control (ab81032, abcam). Goat anti-rabbit Alexa fluor 488 (A11034), Goat anti-rabbit Alexa fluor 568 (A11011), Goat anti-rabbit Alexa fluor 405 (A31556), Goat anti-rabbit Alexa fluor 647 (A21245). Goat anti-mouse Alexa fluor 405 (A31553), Goat anti-mouse Alexa fluor 488 (A28175), Goat anti-mouse Alexa fluor 568 (A11031), Goat anti-mouse Alexa fluor 647 (A21235). Dilutions used for applications are provided in the methods section. |
| Validation | Immunoblotting antibodies: Molecular weight for specificity<br>MAP1LC3B (NB100-2220, NovusBiological): Manufacturer's validation statements: Genetic Strategies: Western Blot, Immunocytochemistry/Immunofluorescence, Immunohistochemistry-Paraffin, Knockdown Validated", Biological strategies: Multiple cell lines in multiple species. It has been cited in previous publication from our lab. PMID: 26541268<br><br>GAPDH (sc-48167, Santa Cruz Biotechnology): Manufacturer's statement: Data from Western Blot, Immunofluorescence, Immunohistochemistry-Paraffin. No validation statement provided This has has been mentioned in large no of citation including PMID: 26541268<br>.COX2 (ab62331, Abcam): Manufacturer's statements: Tested for WB; knockdown validated in lab (mentioned in this manuscript, Supplementary Fig 6E).<br>ICC/ Confocal antibodies:<br>Rab5 (sc-309, Santa Cruz Biotechnology): Manufacturer's statement: Data from Western Blot, Immunofluorescence/flow |

cytometry, Immunohistochemistry-Paraffin. No validation statement provided. Ref to publication from lab: PMID: 26541268

Rab7 (sc-6563, Santa Cruz Biotechnology): Manufacturer's statement: Data from Western Blot, Immunofluorescence/flow cytometry, Immunohistochemistry-Paraffin. No validation statement provided. Ref to publication from lab. PMID: 26541268

LAMP-1 (sc-20011, Santa Cruz Biotechnology). Manufacturer's statement: Data from Western Blot, Immunofluorescence/flow cytometry, Immunohistochemistry-Paraffin. No validation statement provided.
CathepsinD (ab19555, Abcam): Manufacturer's statements: Tested for ICC/IF, IHC-P in humans. No validation statement provided.
Immunofluorescence (IFA)/ Flow cytometry antibodies:
ABCC1 (sc-18835, Santa Cruz Biotechnology): Manufacturer's statement: Data from Western Blot, Immunofluorescence/flow cytometry, Immunohistochemistry-Paraffin. No validation statement provided. Knockdown by siRNA was validated in lab.
ABCG2 (sc-58222, Santa Cruz Biotechnology): Manufacturer's statement: Data from Western Blot, Immunofluorescence/flow cytometry, Immunohistochemistry-Paraffin. No validation statement provided. Knockdown by siRNA was validated in lab.
CD73-FITC (clone AD2, BD 561254): Manufacturer's statement: Specifically recommended for MSC. Cited in multiple publications; PMID: 16923606
CD11b-PE(BD 557321): Ref to publication: Barclay NA, Brown MH, Birkeland ML, et al, ed. The Leukocyte Antigen FactsBook. San Diego, CA: Academic Press; 1997
CD271 (ab8874, Abcam): Ref to publication: PMID: 31298263
CD44 (MS668P, Thermoscientific): Ref to publication: PMID: 7509720
CD90 (ab181469, Abcam) : Ref to publication: PMID: 28890164
CD105 (ab114052, Abcam): Ref to publication: PMID: 26896856
CD45-APC (clone 30-F11; 559864): Ref to Publication: PMID: 11062533
CD73-BV450 (clone TY/23; 561544) : Ref to Publication: PMID: 9808167
CD11b-APC-Cy7 (clone M1/70; 557657) : Ref to Publication: PMID: 8083537
Ly6G-PE (clone 1A8; 551461) : Ref to Publication: PMID: 8360469
Sca-1-PE-CF594 (clone D7; 562730) : Ref to Publication: PMID: 2493502
CD90-BB515 (clone OX7, 564607) : Ref to Publication: PMID: 1940317
CD44-BV605 (Clone IM7, 563058) : Ref to Publication: PMID: 3093091
CD105-PE (Clone MJ7/18, 562759) : Ref to Publication: PMID: 8194490
Ly6c-BV605 (Clone AL-21, 563011) : Ref to Publication: PMID: 7959903
I-A/I-E-BB700 (clone 2G9, 746086): Ref to Publication: PMID: 1331245
CD11c-PE-Cy7 (cloneHL3, 558079): Ref to Publication: PMID: 7751620
Tissue IHC/IFA
Mouse anti-CD73 (1D7) (ab91086, Abcam): Ref to Publication: PMID: 23776241

Rabbit anti-Ag85B (ab43019, Abcam): Ref to Publication: PMID: 29499041
Mouse anti-105 (SN6) (ab11414, Abcam): Ref to Publication: PMID: 31294637

## Eukaryotic cell lines

Policy information about cell lines

| | |
|---|---|
| Cell line source(s) | THP-1 cell line were obtained from American Type Culture Collection (ATCC, Rockville, MD, USA), Adipose tissue derived mesenchymal stem cells (ADSC) were from (Life Technologies, R7788115) |
| Authentication | We did not perform independent authentication |
| Mycoplasma contamination | Cells were tested negetive for mycoplasma contamination |
| Commonly misidentified lines (See ICLAC register) | No cells used in this study are found in the database of commonly misidentified cell lines. |

## Animals and other organisms

Policy information about studies involving animals; ARRIVE guidelines recommended for reporting animal research

| | |
|---|---|
| Laboratory animals | 4 week old C57bl/6 female mice. Animals were housed in individual ventilated cages in the BIosafety lab (BSL3) maintained at 20-25oC, 30-60% humidity and 12-12 hours of light-dark cycle. |
| Wild animals | The study did not involve wild animals |
| Field-collected samples | The study did not involve Field-collected samples |
| Ethics oversight | The animal studies were approved by the Institutional Animal Ethics Committee (IAEC) at ICGEB. |

Note that full information on the approval of the study protocol must also be provided in the manuscript.

## Human research participants

Policy information about studies involving human research participants

| | |
|---|---|
| Population characteristics | This is not a population based study. Colonic biopsy samples were taken from confirmed intestinal tuberculosis (ITB) patients. Blood samples for PBMC isolation was taken from four healthy volunteers. The mean age of healthy volunteers was ~30 years and there were 2 males. Human lung biopsy samples were taken from seven confirmed pulmonary TB patients, diagnosed earlier. The mean age of the patients was 40 years and there were four males. The patients were naïve to antitubercular therapy. Human lung biopsy samples and control sections were obtained from Department of Pathology, AIIMS, New Delhi. These biopsy samples were taken for diagnostic purpose in these patients with written informed consent. Use of the archived leftover biopsy samples were approved by the institute's EC. Blood samples for PBMCs were taken from healthy volunteers. |
| Recruitment | Healthy volunteers were recruited after it was ascertained that they had no systematic illness and were not on any medication. Patients with Intestinal TB were recruited from the Gastroenterology Clinic at All India Institute of Medical Sciences. The diagnosis of Intestinal TB was made if the colon biopsy showed caseating granuloma or presence of acid-fast bacilli or culture positivity for TB. |
| Ethics oversight | AIIMS, New Delhi and ICGEB, New Delhi |

Note that full information on the approval of the study protocol must also be provided in the manuscript.

## Flow Cytometry

### Plots

Confirm that:

- [x] The axis labels state the marker and fluorochrome used (e.g. CD4-FITC).
- [x] The axis scales are clearly visible. Include numbers along axes only for bottom left plot of group (a 'group' is an analysis of identical markers).
- [x] All plots are contour plots with outliers or pseudocolor plots.
- [x] A numerical value for number of cells or percentage (with statistics) is provided.

### Methodology

| | |
|---|---|
| Sample preparation | Cells were pelleted down at 1000 rpm and blocked in 3% BSA in 1X PBS and incubated with primary antibody (1:100) for 3 hr in blocking buffer followed by incubation with alexa flour 488 conjugated secondary antibody (1:200) for 2 hrs (surface expression). Intracellular expression was assessed after permeabilizing cells with 0.05% saponin, followed by blocking, primary (1:100) and secondary antibody (1:200) incubation.<br>Lung tissue was washed with PBS, chopped in to small pieces followed by addition of 20U/ml DNAses, 1mg/ml collagenase D and incubated for 30 mins at 37C. Cells were passed through nylon mesh to get single cell suspension. The cells were pelleted at 2000 rpm, treated with RBC lysis buffer followed by washing with PBS. Cells were stained with the antibody cocktail of and just before sorting PI was added at 5µg/ml for live/dead staining. |
| Instrument | FACS CANTO II (BD) and FACSARIA FUSION (III) (BD) |
| Software | Flow Jo V10.5.3 software |
| Cell population abundance | Sorting samples at 8-10 million per sample, Acquisition samples at 10,000 per sample |
| Gating strategy | The gating strategy is used to exclude cell debris, aggregates and dead cells. The gating strategy is provided in the Figure 4 |

- [x] Tick this box to confirm that a figure exemplifying the gating strategy is provided in the Supplementary Information.
