# Supplementary material for: Mesenchymal stem cells offer a drug-tolerant and immune-privileged niche to Mycobacterium tuberculosis
Source: Nat Commun. 2020 Jun 16;11:3062. doi: 10.1038/s41467-020-16877-3 (PMC7297998; doi:10.1038/s41467-020-16877-3)
Supplement: Supplementary file 4 — Description of Additional Supplementary Files [file 41467_2020_16877_MOESM4_ESM.pdf]

Description of Additional Supplementary Files

File Name: Supplementary Data 1
Description: Data enlisting gene expression profile of ADSCs upon infection with H37Rv
